# CRISP: a deep learning architecture for GC × GC–TOFMS contour ROI identification, simulation and analysis in imaging metabolomics

**DOI:** 10.1093/bib/bbab550

**Published:** 2022-01-11

**Authors:** Vivek Bhakta Mathema, Kassaporn Duangkumpha, Kwanjeera Wanichthanarak, Narumol Jariyasopit, Esha Dhakal, Nuankanya Sathirapongsasuti, Chagriya Kitiyakara, Yongyut Sirivatanauksorn, Sakda Khoomrung

**Affiliations:** Metabolomics and Systems Biology, Department of Biochemistry, Faculty of Medicine Siriraj Hospital, Mahidol University, Bangkok 10700, Thailand; Siriraj Metabolomics and Phenomics Center, Faculty of Medicine Siriraj Hospital, Mahidol University, Bangkok 10700, Thailand; Metabolomics and Systems Biology, Department of Biochemistry, Faculty of Medicine Siriraj Hospital, Mahidol University, Bangkok 10700, Thailand; Siriraj Metabolomics and Phenomics Center, Faculty of Medicine Siriraj Hospital, Mahidol University, Bangkok 10700, Thailand; Metabolomics and Systems Biology, Department of Biochemistry, Faculty of Medicine Siriraj Hospital, Mahidol University, Bangkok 10700, Thailand; Siriraj Metabolomics and Phenomics Center, Faculty of Medicine Siriraj Hospital, Mahidol University, Bangkok 10700, Thailand; Metabolomics and Systems Biology, Department of Biochemistry, Faculty of Medicine Siriraj Hospital, Mahidol University, Bangkok 10700, Thailand; Siriraj Metabolomics and Phenomics Center, Faculty of Medicine Siriraj Hospital, Mahidol University, Bangkok 10700, Thailand; Metabolomics and Systems Biology, Department of Biochemistry, Faculty of Medicine Siriraj Hospital, Mahidol University, Bangkok 10700, Thailand; Siriraj Metabolomics and Phenomics Center, Faculty of Medicine Siriraj Hospital, Mahidol University, Bangkok 10700, Thailand; Section of Translational Medicine, Faculty of Medicine Ramathibodi Hospital, Mahidol University, Bangkok, Thailand; Research Network of NANOTEC – MU Ramathibodi on Nanomedicine, Bangkok, Thailand; Department of Medicine, Faculty of Medicine, Ramathibodi Hospital, Rama VI Rd., Ratchathewi, Bangkok 10400, Thailand; Siriraj Metabolomics and Phenomics Center, Faculty of Medicine Siriraj Hospital, Mahidol University, Bangkok 10700, Thailand; Metabolomics and Systems Biology, Department of Biochemistry, Faculty of Medicine Siriraj Hospital, Mahidol University, Bangkok 10700, Thailand; Siriraj Metabolomics and Phenomics Center, Faculty of Medicine Siriraj Hospital, Mahidol University, Bangkok 10700, Thailand; Center of Excellence for Innovation in Chemistry (PERCH-CIC), Faculty of Science, Mahidol University, Bangkok, Thailand

**Keywords:** chronic kidney disease, imaging metabolomics, deep learning, bioinformatics, GC × GC–TOF

## Abstract

Two-dimensional gas chromatography–time-of-flight mass spectrometry (GC × GC–TOFMS) provides a large amount of molecular information from biological samples. However, the lack of a comprehensive compound library or customizable bioinformatics tool is currently a challenge in GC × GC–TOFMS data analysis. We present an open-source deep learning (DL) software called contour regions of interest (ROI) identification, simulation and untargeted metabolomics profiler (CRISP). CRISP integrates multiple customizable deep neural network architectures for assisting the semi-automated identification of ROIs, contour synthesis, resolution enhancement and classification of GC × GC–TOFMS-based contour images. The approach includes the novel aggregate feature representative contour (AFRC) construction and stacked ROIs. This generates an unbiased contour image dataset that enhances the contrasting characteristics between different test groups and can be suitable for small sample sizes. The utility of the generative models and the accuracy and efficacy of the platform were demonstrated using a dataset of GC × GC–TOFMS contour images from patients with late-stage diabetic nephropathy and healthy control groups. CRISP successfully constructed AFRC images and identified over five ROIs to create a deepstacked dataset. The high fidelity, 512 × 512-pixels generative model was trained as a generator with a Fréchet inception distance of <47.00. The trained classifier achieved an AUROC of >0.96 and a classification accuracy of >95.00% for datasets with and without column bleed. Overall, CRISP demonstrates good potential as a DL-based approach for the rapid analysis of 4-D GC × GC–TOFMS untargeted metabolite profiles by directly implementing contour images. CRISP is available at https://github.com/vivekmathema/GCxGC-CRISP.

## Graphical Abstract

**Graphical Abstract:**
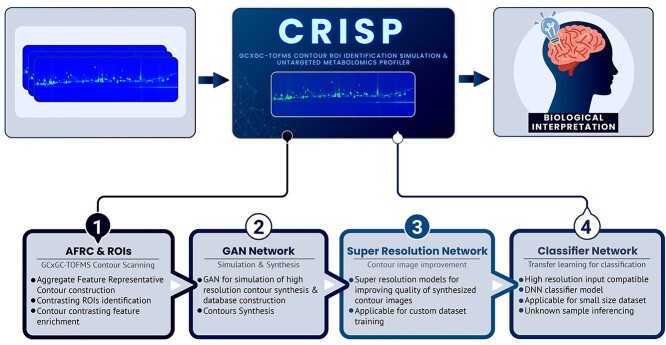

The CRISP software, which combines multiple deep learning models for the interpretation of two-dimensional gas chromatography–time-of-flight mass spectrometry (GC × GC–TOFMS) by directly processing contour images and assisting with the biological interpretation of results.

## Introduction

Gas chromatography–mass spectrometry (GC–MS) is one of the most widely used techniques for metabolic profiling of body fluids because of its high sensitivity, excellent separation performance and availability of databases. It serves as a molecular imaging apparatus and has been used for various medical applications such as metabolic disorder profiling and biomarker discovery for type 2 diabetes mellitus, end-stage renal disease (ESRD) and chronic kidney disease (CKD) [1–4]. Comprehensive two-dimensional gas chromatography–time-of-flight mass spectrometry (GC × GC–TOFMS) is a significant advancement over traditional GC–MS, improving the separation power and identification of molecule constituents in the spatial dimensions while adding a second retention time dimension [5]. GC × GC–TOFMS, when coupled with a high-resolution mass spectrometer, enables detailed analysis of biological fluids with better peak separation and segregation of molecular intensities [6, 7]. The technique has proved to be extremely effective in evaluating trace components in complex mixtures such as human plasma [4, 8]. In particular, a contour image obtained from GC × GC–TOFMS defines the ionized molecular features of high-dimensional chromatogram data as a multichannel two-dimensional image that can be used for rapid analysis of the aggregate sample characteristics [9]. However, limited bioinformatic tools, a lack of standardized protocols for data analysis, and inadequate mass spectral libraries are currently the major challenges faced in GC × GC–TOFMS data analysis. The complex nature of GC × GC–TOFMS and relatively expensive running costs make repeated sample runs difficult for large database generation and analysis. The conventional methods of metabolomics data analysis are also time-consuming and require multiple manual data preprocessing and analysis steps [10]. Furthermore, slight unpredictable shifting in the two-dimensional retention times of each metabolite during chromatographic separation makes it difficult to precisely identify peaks or screen samples using conventional analysis [3]. Given these challenges, advanced computational approaches are necessary for GC × GC–TOFMS data analysis.

Our recent review explored possible applications of deep learning (DL), a branch of artificial intelligence, in metabolomics research [11–13]. DL, particularly in the field of medical technology, has revolutionized the diagnostic aspect of several diseases [11, 14]. DL techniques are now used to predict early stages of cancer as well as to simulate and *de novo* synthesize various types of omics data [15, 16]. Generative adversarial networks (GANs) and convolutional neural networks (CNNs) have been applied in several areas of omics data analysis, including high fidelity sparse sample data simulation [17], gene expression simulation [18], single nucleotide polymorphism-based classification [19, 20], genome-wide association studies [21, 22] and alphafold protein folding prediction [23]. Typically, a GAN is a DL model that can learn and generate entirely new data with the same statistical distribution as its corresponding training dataset. [24, 25] A CNN is the class of deep neural network most commonly used to analyze image features [26]. A region of interest (ROI) refers to any region within the GC × GC–TOFMS contour image with contrasting features that could be used to classify the corresponding sample. Previously, non-negative matrix factorization was used for unsupervised direct GC × GC–TOFMS contour classification [27], but the method was unable to identify multiple ROIs within contour data and its classifier could not be customized. Furthermore, DL has not yet been used for automated multi-ROI identification, simulation, and untargeted profiling of GC × GC–TOFMS contour data.

Here, we present the open-source cross-platform software contour ROI identification, simulation and untargeted metabolomics profiler (CRISP). We demonstrate the potential utility of this integrated DL approach for classifying GC × GC–TOFMS contour images of late-stage diabetic nephropathy and healthy control samples. The CRISP software can also be used to assist in the rapid screening of GC × GC–TOFMS contour image data.

## Material and methods

### Participants and plasma samples

Participants were enrolled by nephrologists at Ramathibodi Hospital, Mahidol University, Thailand. Written informed consent was obtained from the participants before the start of the study. The study was approved by the Ethical Clearance Committee on Human Rights Related to Research Involving Human Subjects, Faculty of Medicine, Ramathibodi Hospital, Mahidol University (COA. MURA2014/369), and all methods were carried out in accordance with the Declaration of Helsinki.

The samples were categorized into a healthy control group (CON; *N* = 20) or a group consisting of ESRD patients on hemodialysis or continuous ambulatory peritoneal dialysis for more than three months with diabetes mellitus (ESRD/DM, *N* = 20). The control group consisted of both male and female subjects with normal renal function, normal urinalysis, and no history of diabetes. Subjects with co-morbidities such as hypertension and those on medication for cardiovascular disease or cancer were excluded from the study.

### Chemical standards and reagents

Hexane, methanol (MeOH), methoxyamine hydrochloride (MeOX), N-methyl-N-(trimethylsilyl)-trifluoroacetamide (MSTFA) + 1% chlorotrimethylsilane (TMCS) and N-tert-butyldimethylsilyl-N-methyltrifluoroacetamide (MTBSTFA) were purchased from Sigma-Aldrich (St. Louis, MO, USA). The stable isotope-labeled internal standards (IS) DL-alanine-3,3,3-d_3_ and L-phenylalanine-1-C_13_ were purchased from Sigma-Aldrich (St. Louis, MO, USA) and Cambridge Isotope Laboratories, Inc. (Frontage Rd, MA, USA), respectively. Pyridine was purchased from Tokyo Chemical Industry, Inc. (Tokyo, Japan). MeOX solution (15 μg/μL in pyridine) was freshly prepared before analysis.

### Sample preparation and GC × GC–TOFMS analysis

The sample preparation for GC × GC–TOFMS analysis was adapted from a previous protocol [28] with minor modifications. In brief, 100 μL of plasma sample was precipitated in 900 μL of pre-cooled 90% aqueous MeOH containing ISs of DL-alanine-3,3,3-d_3_ and L-phenylalanine-1-C_13_ at 20 ng/μL. The mixed solution was left at −20 °C for 1 h and centrifuged at 19 600 g (4 °C) for 10 min. After centrifugation, 200 μL of the supernatant was transferred to a new Eppendorf tube (1.5 mL) and then completely dried in a Centrivap concentrator (Labconco) at 65 °C (~2 h). The sample was kept at −20 °C until analysis. The dried sample was derivatized by methoximation followed by trimethylsilylation (TMS). Briefly, 30 μL of (15 μg/μL) MeOX in pyridine was added to the dried sample, sonicated at 25 °C for 3 min, and incubated at room temperature for 16 h. Subsequently, the mixture was mixed with 30 μL of MSTFA with 1% TMCS and sonicated for 3 min at room temperature. The mixture was incubated at 70 °C for 1 h and transferred into a GC vial for GC × GC–TOFMS analysis. The pooled sample was used as the quality control [29] and was prepared by combining 200 μL of supernatant from each sample and performing the same protocol for sample derivatization described above. The pooled samples were distributed along the run order (every 15 samples).

The derivatized samples were analyzed by GC × GC–TOFMS (Pegasus 4D HRT, Leco Corp. Inc.). The first GC column was a non-polar Rxi-5sil MS column (30 m length, 0.25 mm ID, and 0.25 μM film thickness, Restek, Bellefonte, PA, USA). The second column was a Rxi-17sil MS column (1 m length, 0.25 mm ID, and 0.25 μM film thickness, Restek, Bellefonte, PA, USA). One microliter of the derivatized sample was injected into the GC × GC–TOFMS using a split ratio of 1:20 and an inlet temperature of 250 °C. The GC × GC–TOFMS oven temperature was initially set at 50 °C (5 min hold), and ramped to 180 °C at 25 °C/min (1 min hold), to 220 °C at 10 °C/min (1 min hold), to 260 °C at 15 °C/min, and to 300 °C at 15 °C/min (4 min hold). The modulator period was set to 4 s, with hot and cold pulse durations of 0.8 and 1.20 s, respectively. Helium was used as a carrier gas with a flow rate of 1 mL/min. The GC × GC–TOFMS contour images were acquired using ChromaTOF (Version: 5.50, Leco Corp. for Windows) and then processed by the CRISP software. All samples were run at least twice to obtain contour image data with both low and high column bleeding to evaluate the ability of CRISP to handle dataset variation. The GC × GC–TOFMS contour images generated from the ChromaTOF were used as source images for the development and validation of the CRISP software (Supplementary Figure S1). Two sets of contour images with high and low column bleed were created by running the same samples 10 months later to evaluate the analytical performance potential of CRISP in the presence of experimental instrumental variation. Validation datasets were created by randomly selecting approximately 15% of the original GC × GC–TOFMS contour images from each group.

### Software architecture

**Figure 1:**
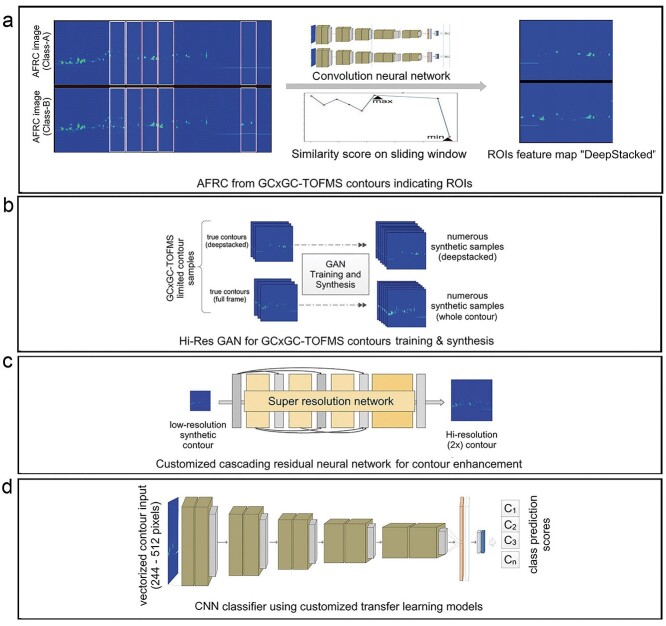
Software architecture. CRISP has four major components. (**A**) The ROI and deepstacking module take contour data input and identifies the ROIs. It then stacks the ROIs using feature detecting CNNs to prepare the datasets. (**B**) The integrated GAN takes the pre-processed data and trains generative models to synthesize high resolution contour image data within the given distribution of the source dataset. (**C**) The contour super-resolution module helps improve the contour image quality prior to use in DNN classifier training. (**D**) The CNN classifier and inference module is customized for 244 × 244 to 512 × 512 pixels input resolutions. It implements multiple transfer learning architectures for training on the contour datasets produced by (**B**) and (**C**). The trained CNN classifier can be used for the subsequent inference of unknown contour profiling. CNN, convolutional neural network; GAN, generative adversarial network; ROI, region of interest.

CRISP consists of four major modules (Figure 1A–D): contrasting feature identification (Figure 1A), contour simulation (Figure 1B), resolution enhancement (Figure 1C) and transfer learning-based GC × GC–TOFMS contour image classification (Figure 1D). CRISP takes GC × GC–TOFMS contour images generated from the ChromaTOF as input data for training or classification and produces report files and watermarked contour images indicating the inference results for unknown samples. The first module of CRISP identifies and enhances the contrasting features between different groups (Figure 1A). It is followed by the second module that simulates contour images (Figure 1B) and the third module that improves the contour image resolution (Figure 1C) to train a classifier for the inference of unknown samples (Figure 1D) and maximize classification efficacy. Although the data generated from GC × GC–TOFMS are multidimensional, the contour images provide a direct glance into the overall feature space of the sample content. Thus, we explored the possibility of profiling all the contour plots with respect to their holistic features, which forms the basis of CRISP’s untargeted metabolite profiling. For contour feature-based untargeted metabolite profiling while avoiding spatial-dimension complexity, CRISP processes the GC × GC–TOFMS contour images in the four modules, described in detail below.

### Module I: Aggregate feature representative contour and ROI stacking

**Figure 2:**
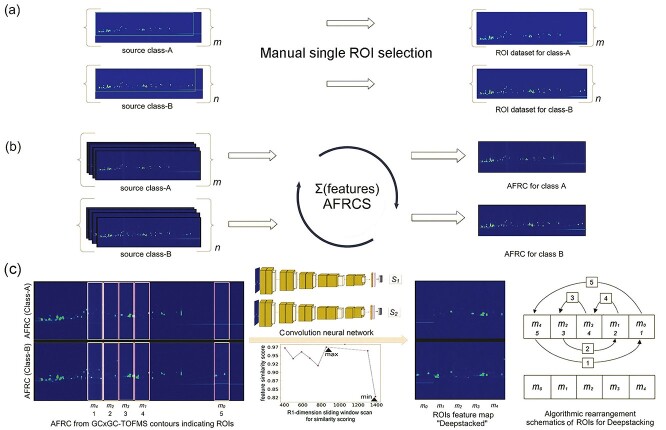
ROI and deepstacking module. This CRISP module is designed to preprocess contour image data for enriched feature dataset construction that can be used by the downstream generative or classifier modules. (**A**) Manual selection of a single ROI to construct a contour dataset. (**B**) Construction of single AFRC image for each study group. (**C**) Semi-automatic identification of multiple ROIs between two classes of contours using VGG16-based feature computation method with example of five identified ROIs (m_1_ – m_5_) stacked algorithmically in ascending order of feature similarity scores to produce a deepstacked dataset. AFRC, aggregate feature representative contour; ROI, region of interest; VGG16, visual geometry group-16.

CRISP introduces the concept of aggregate feature representative contours (AFRCs) for creating a single representative GC × GC–TOFMS contour image for each study group, regardless of differences in sample size among the study groups. The first module directly uses GC × GC–TOFMS contour images from the ChromaTOF (Leco, MI, USA) as input. The software provides an option to manually select a single ROI (Figure 2A) or construct an AFRC image (Figure 2B) for stacking multiple ROIs algorithmically to construct a contour image dataset (Figure 2C). In addition, a single ROI can be selected from the whole contour image for the inclusion of all features. In most cases, the discriminating features are relatively small and dispersed across the entire contour plot. To amplify these sparse contrasting features and minimize manual selection bias, the module uses a novel extraction procedure to construct a single AFRC image for each group. The AFRC image construction approach provides an algorithmic means of representing generalized GC × GC–TOFMS contour feature of a study group. The process is unsupervised and does not discriminate between noise and signal content, resulting in an unbiased representative contour image for each group in the dataset. The AFRC is based on the accumulation of features from the cyclic-ordered rotation of high-resolution contour image content captured at a fixed viewpoint for further foreground–background segmentation. The sequence of image data at a fixed frames per second (FPS) rate is generated by running the source contour images in an iterative manner. Thus, a video is temporarily created using the contour images as frames, and a single aggregate feature of the video content is extracted as an AFRC image. The feature extraction process includes a weight accumulation factor *α* that specifies the amount of information that an AFRC image retains of its previous input image during a cyclic run at a constant FPS. Because the function in OpenCV (https://opencv.org/) by default supports multi-channel image data, each channel is processed independently, preserving the color intensity information in the contour image data. The function is expressed as follows:

(1) \begin{align*} \mathrm{afrc}\left(x,y\right)\leftarrow &\left(1-\alpha \right)\times \mathrm{afrc}\left(x,y\right)\nonumber\\ &+\alpha \cdot \mathrm{sources}\left(x,y\right)\mathrm{if}\kern0.17em \mathrm{content}\left(x,y\right)\ne 0 \end{align*}

Here, sources denotes the set of multichannel input image frames in a cyclic order at a given fixed frame rate, afrc denotes the destination accumulator AFRC image, which has the same number of channels as the input image, and parameter *α* is as described above. The content condition requires the input contour image data to exist.

This process creates a single AFRC that captures the general features of the entire contour content belonging to the group (Figure 2B). The AFRC content is proportionally influenced by the frequency of the contour feature content and automatically suppresses any outlier features during construction. These AFRCs are subsequently processed using a novel stacking approach to create the feature-enhanced GC × GC–TOFMS contour dataset. The CRISP, for the first time in metabolomics, introduces the concept of feature-enriched GC × GC–TOFMS contour image dataset by stacking regions with major differences between the study classes. The CNN-based model is utilized to compute similarity between two AFRCs due to their ability to recognize complex patterns in images. In brief, the features of the AFRCs for any two groups are compared *via* a fixed size scanning window along the first dimension of retention time. A high performance CNN-based image feature extraction model (VGG16) [26, 30–32] was implemented using the default architecture to evaluate the differences in each window and compute the similarity scores. According to these scores, the corresponding ROIs for all source contours are sorted in ascending order and stacked to create a contrasting feature-enhanced dataset called a ‘deepstacked’ dataset (Figure 2C). CRISP stacks the first five ROIs by default with the minimum similarity scores to ensure at least three major ROIs are incorporated in the deepstacked database. In addition to the standard VGG filter, the user can experiment with several alternative similarity metrics such as the Hamming distance [33], PSNR [34], Fréchet inception distance (FID) [35] and SSIM [36] for ROI comparison. This dataset can either be directly sent to the classifier or processed by the GAN synthesizer module.

### Module II: GAN training and contour synthesis

**Figure 3:**
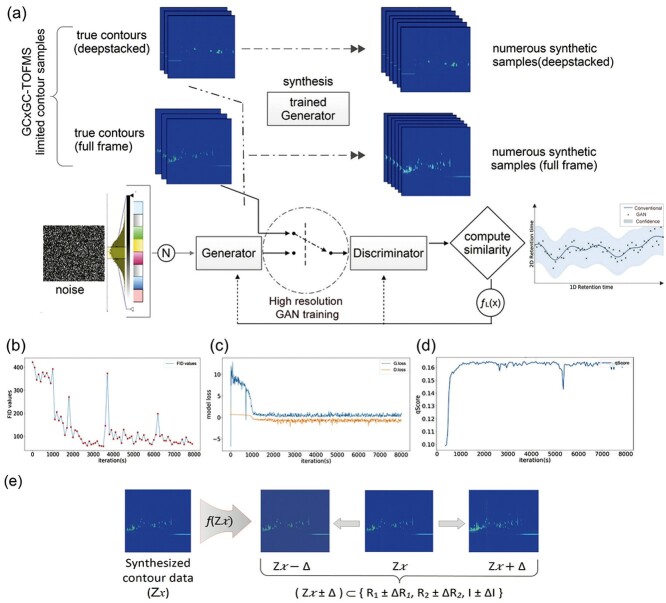
Integrated GAN module. This CRISP module consists of a customizable integrated generative model based on a QP-GAN [37]. (**A**) The dataset containing deepstacked or original full-frame source contour images is supplied to the GAN network, which trains the generator to synthesize corresponding high resolution (256 × 256–512 × 512 pixels) synthetic contours within the source data distribution. Here, ƒL(x) represents FID or a similar scoring metric to compute the similarity of synthesized contour during simulation with the true contours. Once the generator is sufficiently trained, it is able to synthesize numerous contour images independently. Changes in (**B**) FID (**C**) model loss and (**D**) qScore values for synthesized contours images during GAN training. (**E**) Manipulation of the latent space *Z*-vector for intensity variation during contour synthesis. Content inside the dotted circle represents the core engine of the integrated PQ-GAN networks. GAN, generative adversarial network; GC × GC–TOFMS, gas chromatography time of flight mass spectrometry; QP-GAN, quadratic potential generative adversarial network; FID, Fréchet inception distance.

The GAN module consists of the core engine used to generate synthetic GC × GC–TOFMS contour data based on a limited number of true samples (Figure 3A). CRISP uses a modified version of the efficient quadratic potential (QP)-GAN [37]. This GAN architecture minimizes the vanishing gradient and Lipschitz constraint, in contrast to previous GAN algorithms. [37, 38] The model ignores probability divergence and directly converges the probability distributions into source sample distributions, iterating to achieve a min–max optimization with respect to generator loss.

(2) \begin{align*} G,T=&\arg{\min}_G\;\arg{\max}_T{E}_{x\sim p(x)}\left[\log\;\sigma \left(T(x)\right)\right]\nonumber \\ &+{E}_{x=G(z);z\sim q(z)}\left[\log \left(1-\sigma \left(T(x)\right)\right)\right] \end{align*}

For a fixed *T*, the goal of *G* can be represented as

(3) $$ G=\arg{\min}_G\;{E}_{x=G(z),z\sim p(x)}\left[\log \left(1-\sigma \left(T(x)\right)\right)\right] $$

Here, *G* and *T* represent the generator and trainer, respectively, and *σ* is a sigmoid function such that *σ* (*x*) = 1/(1 + *e*^–*x*^) has a loss function of –log *σ* (*T*(*x*)). Furthermore, *Z*, argmax and argmin represent the latent space vector, maxima and minima of functions *G* or *T* for any given input dataset [37]. The QP-GAN adjusts the loss of generator *G* for better optimization instead of focusing on the original min–max game. The FID metric is computed to evaluate the distance between the feature vectors of the simulated and source images to assess the quality of contour image synthesis. [35, 37, 39, 40] Lower FID scores indicate better quality in the synthesized contour images. In the synthesizer training step, the batch size for FID score evaluation can be set and the vector shape can be customized for random noise input. CRISP introduces a novel qScore to estimate the quality of synthesized contour based on image sharpness. The qScore matric evaluates the synthesized image blurriness with respect to the source contour images. For a synthetic contour image, this is calculated as qScore = sigmoid (${\nabla}^2$ (image)), which is the value obtained from a sigmoid function applied to the output of a Laplacian (${\nabla}^2)$ operator. [41, 42] A qScore of 0.18 indicates that the sharpnesses of synthesized and source images are approximately equal. These indicators and other customization features provide real-time visualizations of the FIDs (Figure 3B), generator (G Loss) and discriminator (D Loss) loss functions (Figure 3C), qScores (Figure 3D) and a user-defined preview of a simulation image grid for visually inspecting the GAN model training status. The contour synthesizer is a well-trained generator *G* that can synthesize image-wise unique contours within the given data distribution and can be customized to obtain various intensity levels for each synthetic contour (Figure 3E). This was done by proportionally manipulating the latent space-associated vector *Z* for each noise input in the trained synthesizer. The GAN module consists of multiple image preprocessing and augmentation features that include contour sharpening, blurring, noise/denoising, erosion, dilation, distortion, brightness, contrast, and edge-enhancement filters for both model training and synthesis. Once properly trained, the synthesizer can be used independently to generate random contour images within the distribution of the training dataset (Figure 3A). The full and deepstacked contour images were constructed using their corresponding trained synthesizers to generate datasets that are ten times larger than the datasets of the original source images.

### Module III: Super-resolution network

**Figure 4:**
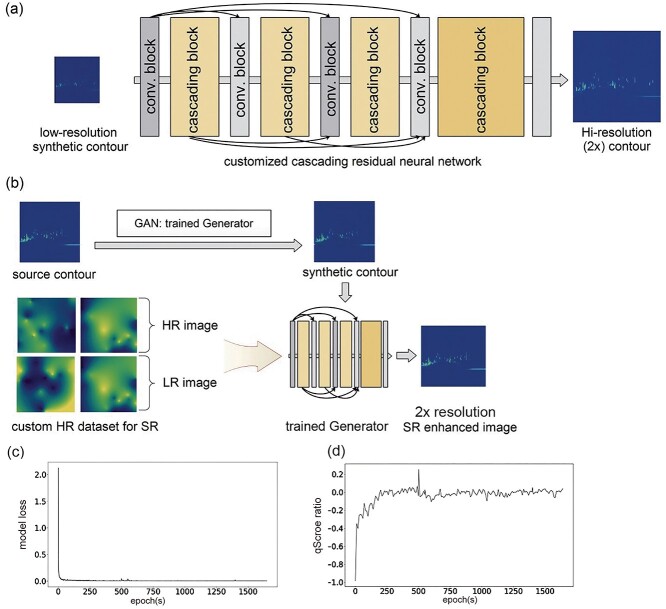
Contour super-resolution module. This CRISP module is based on CARN [43] to improve contour image resolution. (**A**) The architecture of CARN, showing the multiple layers of network responsible for image resolution improvement. (**B**) The super-resolution network is specifically trained using high quality contour-like features similar to those in the simulated dataset. The network is trained until the (**C**) model loss and (**D**) qScore ratio are sufficiently low. The trained model can be used independently to improve the quality of contours synthesized by the generator module. CARN, cascading residual network, HR, high resolution; LR, low resolution, GAN, generative adversarial network.

CRISP implements a cascading residual network (CARN)-based super-resolution CNN to enhance the resolution of the relatively low-resolution contour images [43]. The contour image synthesized by the GAN module can be optionally processed by the CARN module to enhance image resolution without compromising output quality and speed (Figure 4A). This super-resolution model was reported to outperform SelNet, DRCN and SRDenseNet in terms of computational cost and performance [43]. The CARN network is trained on high-quality GC × GC–TOFMS contour images to optimize the super-resolution model and hence improve the quality of the synthesized contour images. Because GC × GC–TOFMS contour images resemble a mosaic or have a plasma-like appearance, a custom simulated high-quality contour-like dataset was constructed to train the contour-specific super-resolution network (Figure 4B). The CARN module employs the L1-loss as a model loss function, which is the mean of the absolute difference between the predicted and true resolution images during training. The L1-loss was reported to yield better convergence and performance than the regular L2-loss, which is the mean-squared error [43]. CRISP also introduces the novel qScore ratio to view the real-time changes in image sharpness during training. This score is computed as the difference in image sharpness of the high- and low-resolution contour images divided by the sharpness of the high-resolution contour image during CARN model training. A qScore ratio of zero indicates approximately equal levels of sharpness in a super-resolution enhanced image and its corresponding original high resolution source image.

### Module IV: Classification network (transfer learning-based contour classifier)

**Figure 5:**
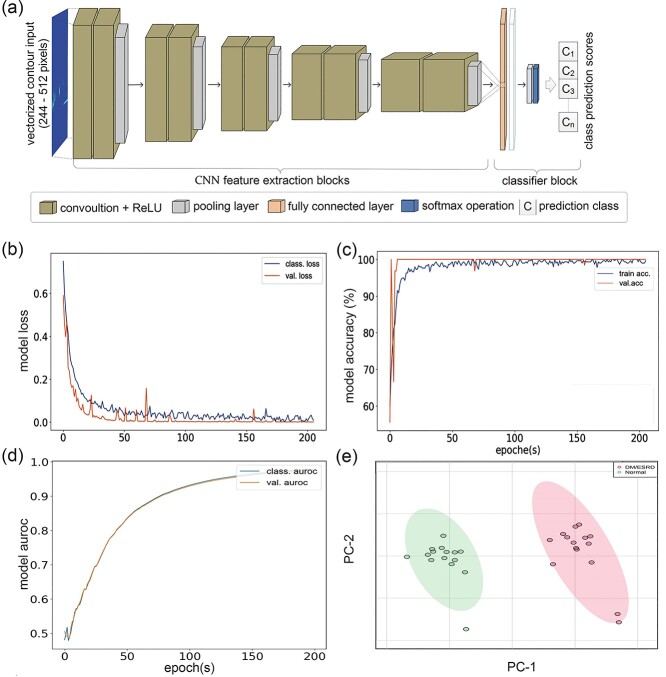
CNN classifier and inference module. The deep CNN is the core engine of CRISP and utilizes transfer learning for feature extraction and classification on the pre-processed dataset. (**A**) Multilayer architecture of the transfer learning-based CNN responsible for feature computation and scoring. The transfer learning model’s (**B**) loss (**C**) accuracy, and (**D**) AUROC for evaluating performance of the CNN classifier. (**E**) A conventional PCA analysis plot for the GC × GC–TOFMS data showing the contrasting features between the ESRD and CON groups. AUROC, area under the receiver operating characteristic; CNN, convolutional neural network; GC × GC–TOFMS, two-dimensional gas chromatography time-of-flight mass spectrometry.

The final CRISP module uses customizable CNN models capable of increasing image input size from 128 × 128 to 512 × 512 pixels for transfer learning-based classification of the GC × GC–TOFMS contour image data. The dataset made from the original, synthesized or combined contour images can be supplied to this module for training the classifier CNNs. The CNNs available in CRISP for transfer learning consist of VGG16 [26], VGG19 [44], Inception V3 [45], RasNet, [46] and DenseNet [47], which enable high-fidelity contour feature classification [44, 48]. To maximize the feature content input to train the classifier models, the input resolution was increased to 512 × 512 pixels instead of the default 244 × 244 pixels. This enables the models to process more information from the source contour images (Figure 5A). To simplify the interpretation and optimize CRISP, VGG16 was used as the default transfer learning-based CNN model for contour image classification. The transfer learning module of CRISP has two submodules.

The first submodule is the classifier trainer, which implements transfer learning using an input contour dataset and can handle multiple classes. The datasets are separated into training and validation sets for evaluating both the training and validation accuracies, model losses, and area under the receiver operating characteristic (AUROC) values. In particular, the AUROC metric quantifies the overall performance of the classifier model in terms of sensitivity and specificity. The training submodule has a built-in image augmentation option, which performs additional multiple random image augmentation operations (e.g., image shearing, skewing and distortion) to increase diversity in the training dataset.

The second submodule is contour inference, and it is the final process in the CRISP software. This submodule uses the trained classifier model to infer unknown contour samples at a pre-defined confidence threshold (set to 85%) and predict their classes. Prior to inference, the preprocessing of the contour image input for whole or deepstacked images should be matched to the type of dataset used to train the classifier model. To interpret results, a customizable report file is generated containing the class prediction and the corresponding contour images tagged with prediction scores. The classification accuracies of conventional machine learning classifiers (decision trees, K-nearest neighbors, random forests, support vector machines, linear regression and simple artificial neural networks) and Keras-based standard DL models (https://keras.io) were compared with the classification accuracy of CRISP. The conventional classifiers were computed using the Scikit-learn machine learning package [49]. The conventional approach of principal component analysis (PCA) was used to validate the raw GC × GC–TOFMS datasets before they were used to optimize the DL software [50].

### Model configuration, logging and updates

CRISP has many settings for GC × GC–TOFMS contour ROI extraction, deepstacking, simulation, resolution enhancement and contour classification. In addition, each trained model has its training history configuration, which includes iteration counts, model loss, accuracies, FIDs, AUROCs and other metrics. The settings needed to run each module can be stored as a plain text configuration file to restore, edit, or directly execute the program using the graphical user interface (GUI) or command-line interface. All model weights and associated ROI and AFRC settings can be stored so that the same ROIs can be used for the inference of similar contour images in future. A plain-text summary of information including the model type, input dimension, trained iteration/epoch, source dataset location and loss function is stored along with the trained weights to provide a quick overview of the model history. The platform can also store logs of most activity to assist error tracking and troubleshooting. Likewise, the updated or pre-trained models for each module, which are stored in Google drive or on an HTTP server, can be manually downloaded and used through a built-in feature of CRISP. Interested researchers can also submit their custom trained models or datasets with a proper description and be listed for download on the CRISP official web repository after manual review.

### Computational hardware and DL software platform

The construction and computation of all CRISP DL models were completed using an Intel core i9 processor with an NVIDIA RTX3070 series CUDA-core compatible graphics processing unit with 8 Gb VRAM and 32 GB system onboard DDR5 RAM. The entire cross-platform compatible software was written in Python and TensorFlow-backend Keras application programming interface. [51, 52] The GUI was designed using PyQt5. Pre-trained base weights for the CNN models (VGG16, VGG19, ResNet50, DenseNet and InceptionV3) used in the transfer learning-based classifier were downloaded from the official Keras website (https://keras.io/api/applications/#available-models). CRISP features both GUI and command line interface architecture for novice to advanced level customization. The updates and trained DL models of CRISP can be directly downloaded from its GUI interface.

## Results and discussion

Initially, to develop CRISP, we used 15 contour images from both the ESRD/DM and CON groups to train the CRISP architecture. We used 3–5 images depending upon the quality of the datasets from both groups for model validation. Furthermore, we tested how the variation in analysis affects the model performance by repeating the analysis for GC × GC–TOF measurements using the same source samples (*N* = 20 for each group) after ten months had passed.

### ROIs, AFRC and deepstacking

**Figure 6:**
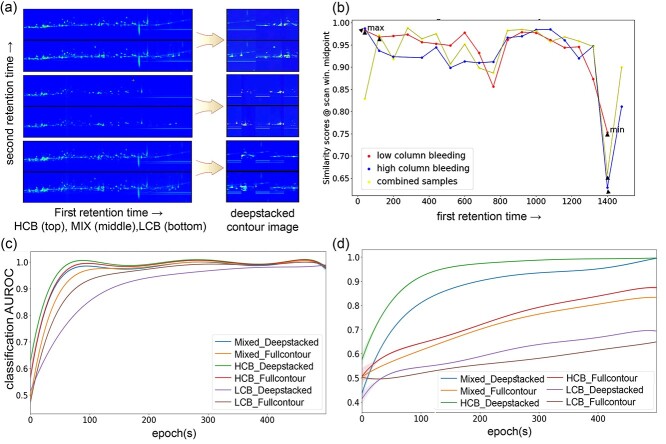
Comparison of ROIs and deepstacking for samples with different amounts of column bleeding. The HCB and LCB GC × GC–TOFMS contour datasets were obtained by analyzing the same samples with the same experimental setup again after an interval of 10 months. (**A**) The ARFC image and ROI deepstacking for the HCB (top), MIX (middle) and LCB (bottom) datasets using the original GC × GC–TOFMS contour images. The deepstacked feature map is constructed by stacking the top five least similar scoring ROIs. (**B**) Full sliding-window feature similarity scores for multiple ROIs based on VGG16 filter for low, high and combined column bleeding contour datasets using the default settings. Classification AUROCs for classifiers trained on full and deepstacked contours for the (**C**) high resolution (512 × 512 pixels) and (**D**) low resolution (244 × 244 pixels) trained models to compare classifier performance at different levels of column bleeding and model resolution. LCB, low column bleed; HCB, high column bleed; MIX, a mixture of LCB and HCB.

The first module of CRISP was designed to take the GC × GC–TOFMS contour images of samples directly from the ChromaTOF and construct the feature-enhanced deepstacked dataset. The contour images from the ESRD/DM and CON groups were separately processed to generate AFRC (Figure 2B) images and the five most contrasting ROIs were identified to construct a deepstacked dataset (Figure 2C and Supplementary Figures S2–S4). Although contrasting features are often small and distributed throughout contour images, CRISP was able to identify major ROIs that were consistent with the overall differences observed from conventional metabolomics data analysis. For instance, the ROIs indicated regions with aberrantly high levels of metabolites such as maltitol, mannitol, sorbitol and dulcitol, which were the most contrasting metabolites observed in the PCA results of the ESRD/DM and CON groups (Figure 5E and Supplementary Figure S11). In contrast to processing an entire contour image or a single ROI (Figure 2A and Supplementary Figure S2), the construction of a deepstacked dataset for each group enabled the features to be enhanced by algorithmic means while minimizing manual selection bias (Figure 2B and C and Supplementary Figures S3 and S4). It was possible for the minor ROIs that might have been otherwise ignored during manual selection to be proportionately represented in the AFRC images for each class. The effect of any existing outlier contour image features was likely diminished because of their low occurrence among the total samples during AFRC construction. The creation of an AFRC image for ROI identification and deepstack database construction might be a suitable way to enhance features in small to medium contour image datasets. Column bleed and a slight shift in retention time are the most common issues that affect data quality and analysis in gas chromatography [53–55]. Experiments involving contour dataset construction using low, high and mixed levels of column bleed exhibited a similar pattern of AFRC construction. In each case, CRISP was able to handle data variation without compromising the identification of key features in GC × GC–TOFMS contour images (Figure 6A and B). Subsequently, a deepstacked dataset was constructed by incorporating the top five ROIs for the enhancement of contrasting features that could be sent to downstream CRISP modules to improve their classifier performance. The feature-enhanced deepstacked dataset constructed using mixed column bleed samples was used in this case because it represented the highest number of samples without compromising the key features of each test group.

### GC × GC–TOFMS contour image simulation

Generative DL models have the architectural advantage of being able to learn complex features from training datasets and synthesize completely new data with the same distribution. [24, 56] This CRISP module was designed to use the feature-enhanced datasets generated by the previous module to train a generator for creating synthetic GC × GC–TOFMS contour images. The synthesizer module of CRISP facilitated the generation of a 10× increase in the number of similar contour images containing randomly induced variation within the data distribution for each group (Figure 3A and Supplementary Figures S5 and S6). The QP-GAN is more suitable for the CRISP synthesizer module than other GANs because of its implementation of a Lipschitz constraint [38] on the discriminator to prevent the vanishing gradient, retain small source data features and prevent model collapse [37, 38]. This model is more powerful than previous GANs (e.g., WGAN, LSGAN and SGAN) because the synthesized images can be scaled up to 512 × 512 pixels with decent quality, in contrast to the 128 × 128–256 × 256 pixels limitations of previous GANs [37]. Although there is currently no gold standard for directly measuring the quality of GAN-synthesized images, the highly effective FID scores provided unbiased estimates of the contour image likeliness relative to the source datasets [35]. The synthesizer was able to simulate contours with an FID of <47.00 (Figure 3B) while converging the losses of the generator and discriminator (Figure 3C), which correlated with observed decent-quality contours for a relatively small source dataset (Figure 3C and Supplementary Figure S6). The variation introduced by combining the original samples with synthetic images provided a reasonably large pool of samples for training the CRISP classifier module. The custom-trained QP-GAN synthesizer generated images with a resolution of 512 × 512 pixels, which meant that nearly four times the amount of contour image details could be sent to the downstream classifier module when compared with the default input of 244 × 244 pixels (Figure 3A and Supplementary Figure S6). The implementation of QP-GAN may facilitate proper training with a limited sample size, unlike most generative models in clinical research that require large image datasets. [57, 58] The experimental qScore was approximately 0.165, indicating that contour images were synthesized that were similar to the source images in terms of image sharpness (Figure 3D). The qScores together with the FIDs indicate the qualitative traits for feature similarity and image quality during GC × GC–TOFMS contour simulation. The real-time graphs for FIDs, model loss convergence and qScores, calculated for batches of simulated images during the training session, indicated a trend highly similar to the source contour images simulated during model training (Figure 3B–D). Unlike traditional GANs [17], the CRISP provides customizable *Z*-vector enabled manipulation of the features during contour synthesis (Figure 3E), which can simulate contour images with different intensities, which is analogous to the concentration variation in true GC × GC–TOFMS contour data. Because GAN models are relatively hard to train, the real-time indicators are crucial not only to adjust the model hyperparameters but also for minimizing the loss of time and computational resources [25, 37]. CRISP’s optional image-enhancement module uses a CARN-based DL super-resolution model for fast and accurate improvement of synthesized contour image quality (Figure 4A and B and Supplementary Figures S7 and S8) [43]. The custom dataset was able to properly train the super-resolution model based on the observed model loss (Figure 4C) and qScore ratios (Figure 4D and Supplementary Figure S7). Although the use of super-resolution slightly enhances the quality of the synthesized contours, no significant improvements were observed during classifier training. This may be a result of the features of the dataset, quality of the synthesized image, and efficacy of the classifier, which was able to detect contrasting features without the need for super-resolution. However, this function provides users the option to train the model and implement super-resolution on synthesized images for custom datasets.

### Classifier training and inference

The performance of the CRISP classifier model is affected by image resolution because a larger input shape can hold more contour feature information during training (Figure 6C and D). However, training at a higher resolution is memory intensive, time consuming, and limited by the architecture of the CNNs. The customizable GUI feature of CRISP classifier (Supplementary Figure S9) and inference (Supplementary Figure S10) modules makes it easier to train and predict classes, respectively. The 512 × 512 pixels high resolution classifier model trained on the 10× simulated dataset yields promising improvements in model performance within the first 200 epochs (Figure 5B–D). The result is in agreement with the PCA score plot (from conventional data analysis; Figure 5E and Supplementary S11) and confirms the difference between the ESRD/DM and healthy control groups. The clear increases in classification accuracy obtained by CRISP (Table 1) with respect to the accuracy obtained by the conventional histogram-based pixel approach indicates the potential of the transfer learning models in contour image-based metabolomics profiling.

**Table 1:** Comparison of CRISP classification accuracies with conventional histogram-based image profilers and artificial intelligence models

S.N	Model	Image histogram-based accuracy^a^	
		244 × 244 pixels	512 × 512 pixels
1	Direct Tree Classifier	84.14 ± 0.53%	86.00 + 0.45%
2	Extra Tree Classifier	84.54 ± 0.63%	84.00 ± 0.76%
3	K-Nearest Neighbors	70.00 ± 0.45%	70.0 ± 0.54%
4	Random Forest	64.17 ± 1.32%	67.37 ± 1.22%
5	Supervised Vector Machine	62.00 ± 0.35%	65.0 ± 0.75%
6	Linear Regression	62.00 ± 0.35%	30 ± 0.45%
7	Simple artificial neural network (ANN)	62.00 ± 0.36%	68.0 ± 0.55%
8	State-of-art CNNs (Keras DCNN, DenseNet, VGG19/VGG16,InceptionV3)	≥94.00%	N/A^b^
9	CRISP	96.45 ± 0.77%	>98.45%

^a^Full-size contour for the mixed column bleed dataset was used for computing classification accuracy. For fetching GC × GC–TOFMS contour image histogram data to machine learning models, input images were resized to 244 × 244 or 512 × 512 pixels and a three-dimensional color histogram was extracted from the HSV color space. The values were normalized and flattened to a one-dimensional feature vector which was subjected to different machine learning classifiers. A train-test-split ratio of 85:15 was applied and each classifier model was tested to fit corresponding models and prediction accuracy was computed.

^b^The default input shape for state-of-art CNN is 244 × 244 pixels while CRISP can take up to 512 × 512 pixels RGB image. All neural network and DL models were run for at least 500 epochs to compute prediction accuracies. CNN, convolutional neural networks; GC × GC–TOFMS, two-dimensional gas chromatography time-of-flight mass spectrometry; HSV, hue saturation value.

**Figure 7:**
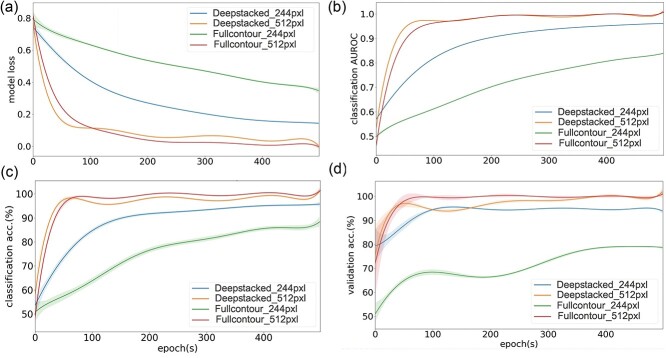
Comparison of classifier performance in terms of (**A**) model loss (**B**) classification AUROC, (**C**) classification accuracy, and (**D**) validation accuracy for GC × GC–TOFMS contour image datasets made using full and deepstacked contour image datasets and trained using a VGG16-based customized transfer learning technique. The high resolution (512 × 512 pixels) and default low resolution (244 × 244 pixels) transfer learning models were separately trained for 500 epochs on the corresponding dataset and trendlines of different indicators are plotted. Shaded regions around the trendlines indicate fluctuation in the data points during training. AUROC, area under the receiver operating characteristic; GC × GC–TOFMS, two-dimensional gas chromatography time-of-flight mass spectrometry.

**Figure 8:**
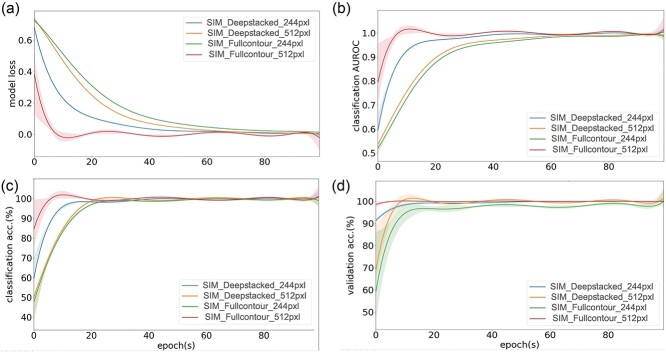
Comparison of classifier performance in terms of (**A**) model loss (**B**) classification AUROC, (**C**) classification accuracy, and (**D**) validation accuracy for the GC × GC–TOFMS contour image dataset, constructed using 10× simulated full and deepstacked contour image dataset and trained using a VGG16-based customized transfer learning technique. The high resolution (512 × 512 pixels) and default low resolution (244 × 244 pixels) transfer learning models were separately trained for 100 epochs on the corresponding dataset and the trendlines of the different indicators are shown. Shaded regions around the trendlines indicate fluctuation in the data points during training. AUROC, area under the receiver operating characteristic; GC × GC–TOFMS, two-dimensional gas chromatography time-of-flight mass spectrometry.

Furthermore, column bleeding, retention time shift and spectrum noise are common problems in GC–MS-based metabolomics analysis [55]. To address these issues related to experimental variation, the source samples were re-evaluated under the same measurement conditions to obtain GC × GC–TOFMS contour datasets with high and low amounts of column bleed. The CRISP classifier model uses an aggregate feature of the contour images, and hence the slightly different contour images obtained from both two experiments conducted 10 months apart had no effect on classifier performance. The full and deepstacked contour datasets derived from the high, low and mixed column bleed source samples all had an AUROC of more than 95.00% (Figure 7C) for the 512 × 512 pixels high resolution classifier models, indicating the relatively good performance of the trained classifier models compared to the 244 × 244 pixels low-resolution classifier models with AUROC values of less than 95%. For the 244 × 244 pixels low resolution dataset, the model trained on the mixed samples dataset had performed better (>85.00% AUROC) than the high or low column bleed datasets (>80.00% AUROC) within 500 epochs. The improvement in performance of the low-resolution trained model on only the mixed dataset might be due to the combined larger sample size (Figure 6C). Thus, the mixed datasets provide a suitable option for incorporating maximum diversity during simulation and classifier training. The 512 × 512 pixels classifier trained on the full and deepstacked datasets had substantially lower model loss (Figure 7A) and higher performance (Figure 7B–D) than the default 244 × 244 pixels-trained model on the non-simulated contour datasets. Similarly, the 512 × 512 pixels classifier trained on the full and deepstacked datasets had substantially lower model loss (Figure 8A) and higher performance (Figure 8B–D) than the default 244 × 244 pixels-trained model on the 10× simulated contour datasets. The classifiers trained with the deepstacked and 10× simulated datasets were able to consistently achieve higher or similar levels of classification performance (Figures 7B and 8B) and model accuracy (Figures 7C and 8C) within one fifth the training epochs used for the non-simulated contour image datasets. The model performance observed for the 512 × 512 pixels deepstacked-trained models exhibited substantial increases in discrimination capacities of classifiers. These models could achieve an AUROC of >0.96 and accuracy of >96.00% within the first 100 epochs, which supports the potential of the CRISP’s ROI-stacking approach to efficiently train contour classifier models with a small sample size. The removal of similar regions among test groups and inclusion of contrasting ROIs during feature enhancement could have mitigated the potential decrease in classifier model efficacy. The 10× increase in the GC × GC–TOFMS training data size obtained by the CRISP synthesizer could potentially cause a reduction in model overfitting and compensate for the lack of a large source dataset. Even though CRISP tries to compensate for issues related to model overfitting by using a 10× simulated dataset, the current limitation in original GC × GC–TOFMS training data size may exert some level of influence on the actual performance of the model. The contour features that could be used as input to classifier models were much smaller in the default CNN models (Figure 5A), with an input shape of 244 × 244 pixels needed to gain good performance. Low-resolution input contour images fundamentally mean information could be lost from the start, which could have affected the classifier performance (Figures 7B and 8B) for the 244 × 244 pixels contours regardless of the differences among the sample classes and transfer learning models used. The deepstacked simulated dataset in combination with a larger input resolution of 512 × 512 pixels achieved the best performance, with an AUROC of >0.95 and accuracy of >96.00%, within the first 100 epochs, which indicates the potential of the CRISP’s approach to efficiently training a contour classifier model with a small available sample size. The training of a classifier with a larger number of source contour images and the corresponding simulated datasets might increase the accuracy of the models.

CRISP could be used to rapidly screen GC × GC–TOFMS contours for pathogen-related disease and healthy control groups if substantial differences appear in the metabolite contents. Some pathogens are known to produce an aberrant and often unidentified collection of metabolites as disease fingerprints in host specimens [59], which could be holistically profiled by CRISP based on their GC × GC–TOFMS contour image. Most of the core DL engines of CRISP are based on image analysis using modified versions of state-of-the-art CNNs. Thus, significant changes in the GC × GC–TOFMS contour profiles could even allow CRISP to predict potential pathogens if the proper training datasets and protocols are provided. Likewise, CRISP is equipped with many options for conducting numerous combinations of experiments that are beyond the scope of the current study. The novel approach of CRISP demonstrates the potential of integrated DL in untargeted GC × GC–TOFMS metabolite profiling that directly implements contour images.

## Summary

The CRISP software uses an integrated DL approach for untargeted GC × GC–TOFMS contour profiling and was evaluated using an in-house GC × GC–TOFMS contour image datasets. The novel approach of AFRC construction combined with ROI stacking helps enhance contour image features for contour profiling in an unbiased manner. The synthesizer module enables the use of a small dataset, highlighting the potential of CRISP to facilitate DL model training for GC × GC–TOFMS datasets with relatively few samples. The fully operational GUI with real-time graphs and model configuration storage features make CRISP easy to operate and allows changes to be tracked. Even though a limited contour dataset and lack of well-established protocols are few limitations of the current version, CRISP may provide profiling scheme for GC × GC–TOFMS data that is an alternative to existing conventional methods.

**Key Points**

- First direct use of contour images in a deep learning approach for GC × GC–TOFMS untargeted metabolite profiling is reported.
- The aggregate feature representative contour image, which automatically identifies contrasting regions between any two groups of GC × GC–TOFMS contour images, is introduced.
- Because of the holistic feature analysis, column bleed and other experimentation variation have little or no effect on contrasting feature identification and classification efficacy.
- A fully operational user interface with real-time indicators and single software pipeline for contrasting feature detection, simulation and classification of GC × GC–TOFMS contour images is provided.

## Supplementary Material

Revision_Supplimentary_information_bbab550

## Data Availability

The source code of CRISP along with information for installation and operation manuals is available in a GitHub repository (https://github.com/vivekmathema/GCxGC-CRISP). The original in-house GC × GC–TOFMS contour images and associated raw data files used for construction and optimization of CRISP will be made available upon reasonable request. The information about CRISP is also made available at http://metsysbio.com/CRISP_HTML/crisp_webinfo.html.

## Notes

**Vivek B. Mathema** has a PhD in bioclinical sciences and is currently a researcher at the Siriraj Metabolomics and Phenomics Center, Faculty of Medicine Siriraj Hospital. Research interest include metabolomics, bioinformatics, oncology, parasitology and molecular epidemiology.

**Kassaporn Duangkumpha** received a PhD in Medical Biochemistry and Molecular Biology. Currently, she is a researcher with an interest in metabolomics for biomedical research.

**Kwanjeera Wanichthanarak** has a PhD in Bioscience. Her main areas of research are systems biology and bioinformatics. She is currently a senior researcher at the Siriraj Metabolomics and Phenomics Center.

**Narumol Jariyasopit** Narumol Jariyasopit has a PhD in Chemistry. Her research focuses on Mass spectrometry based-metabolomics.

**Esha Dhakal** (M. Sc.) is a graduate student at the department of Biochemistry, Faculty of Medicine Siriraj Hospital. Her research interest includes mass spectrometry and biochemistry of diseases.

**Nuankanya Sathirapongsasuti (MD, PhD)** is an instructor at the Section of Translational Medicine, Faculty of Medicine Ramathibodi Hospital. Research interest includes human diseases and health related organisms from multi-omics perspective.

**Chagriya Kitiyakara (MD)** is a professor of Medicine at Faculty of Medicine Ramathibodi Hospital. His research interests are biomarkers and metabolomics in kidney diseases.

**Yongyut Sirivatanauksorn (MD, PhD)** is a director of the Siriraj Metabolomics and Phenomics Center, and the founding president of the Thailand Metabolomics Society. His research interests are Hepatopancretobiliary cancer and liver transplantation.

**Sakda Khoomrung** (PhD in Chemistry) is an instructor at the Faculty of Medicine Siriraj Hospital. His research interest is on the development of tools in metabolomics and systems biology to support translational research.
