# Supplementary material for: CRISP: a deep learning architecture for GC × GC–TOFMS contour ROI identification, simulation and analysis in imaging metabolomics
Source: Brief Bioinform. 2022 Jan 11;23(2):bbab550. doi: 10.1093/bib/bbab550 (PMC8921635; doi:10.1093/bib/bbab550)
Supplement: Revision_Supplimentary_information_bbab550 [file revision_supplimentary_information_bbab550.docx]

**CRISP: A deep learning architecture for GC×GC-TOFMS contour ROI identification, simulation, and analysis in imaging metabolomics**

### Vivek Bhakta Mathema^1,2^, Kassaporn Duangkumpha^1,2^, Kwanjeera Wanichthanarak^1,2^, Narumol Jariyasopit^1,2^, Esha Dhakal^1,2^, Nuankanya Sathirapongsasuti^3,4^, Chagriya Kitiyakara^5^, Yongyut Sirivatanauksorn^2^, Sakda Khoomrung^1,2,5^**^*^**

^1^Metabolomics and Systems Biology, Department of Biochemistry, Faculty of Medicine Siriraj Hospital, Mahidol University, Bangkok 10700, Thailand

^2^Siriraj Metabolomics and Phenomics Center, Faculty of Medicine Siriraj Hospital, Mahidol University, Bangkok 10700, Thailand

^3^Section of Translational Medicine, Faculty of Medicine Ramathibodi Hospital, Mahidol University, Bangkok, Thailand

^4^Research Network of NANOTEC - MU Ramathibodi on Nanomedicine, Bangkok, Thailand

^5^Department of Medicine, Faculty of Medicine, Ramathibodi Hospital, Rama VI Rd., Ratchathewi, Bangkok 10400, Thailand

^6^Center of Excellence for Innovation in Chemistry (PERCH-CIC), Faculty of Science, Mahidol University, Bangkok, Thailand

*Corresponding author: [sakda.kho@mahidol.edu](mailto:sakda.kho@mahidol.edu)

**Source code, models and datasets** (Fully available only after acceptance of manuscript)

The source code and minimal example dataset, models can are available in Github:

Weblink: <https://github.com/vivekmathema/GCxGC-CRISP>

**The manual for CRISP can be downloaded from github:**

Weblink: <https://github.com/vivekmathema/GCxGC-CRISP/blob/main/Manual/CRISP_manual.pdf>


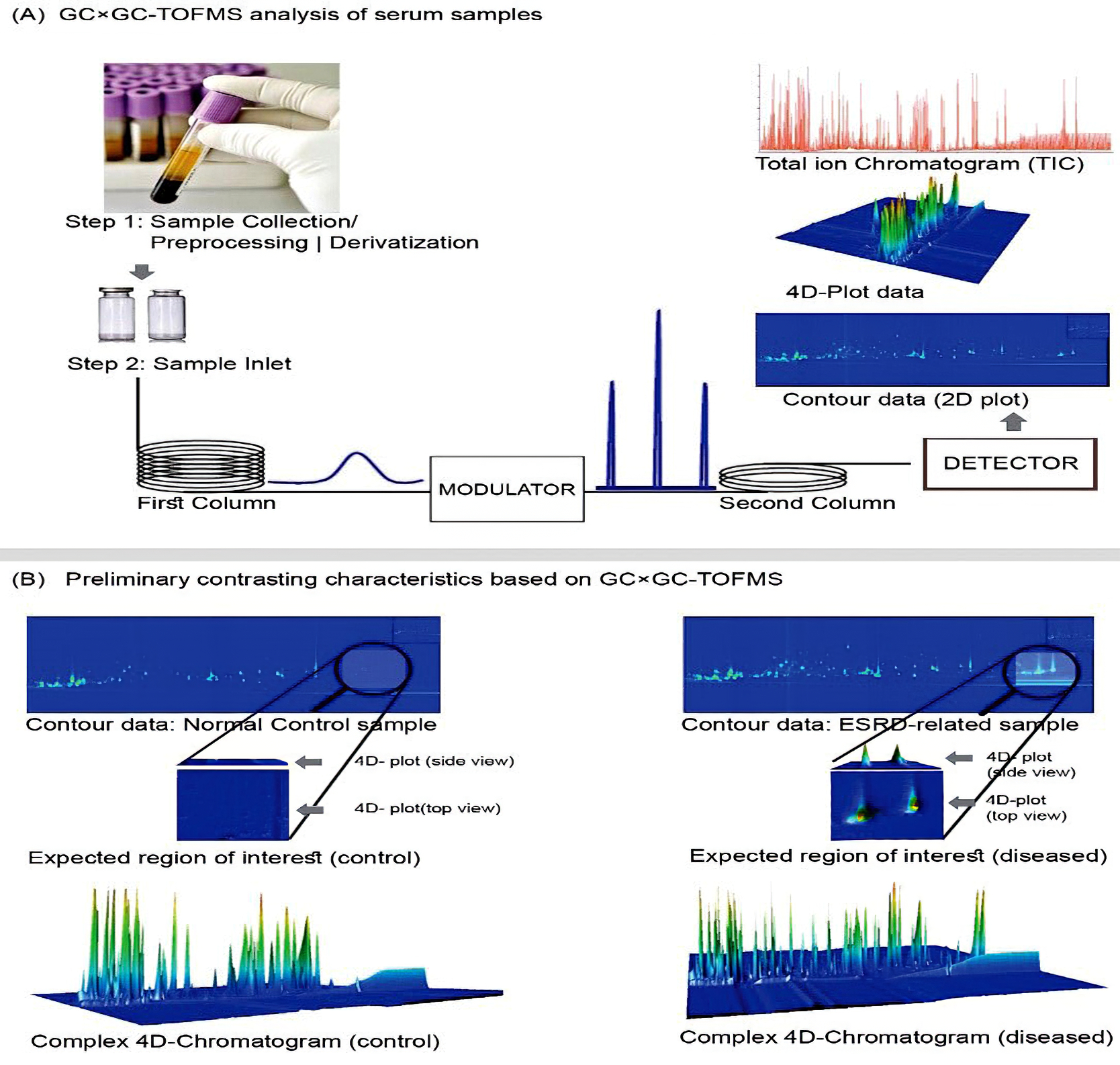


**Figure S1.** General schematics of the GC×GC-TOFMS indicating major steps involved in sample processing to generate contour data for healthy controls and the ESRD/DM group. (A) Processing of the samples in GC×GC-TOFMS to obtain RAW as CDF file. (B) Contour image data from ChromaTOF, showing some major differences between healthy and diseased (ESRD/DM) group. ESRD/DM, end-stage renal disease with diabetes mellitus; GC×GC-TOFMS, two-dimensional gas chromatography time-of-flight mass spectrometry
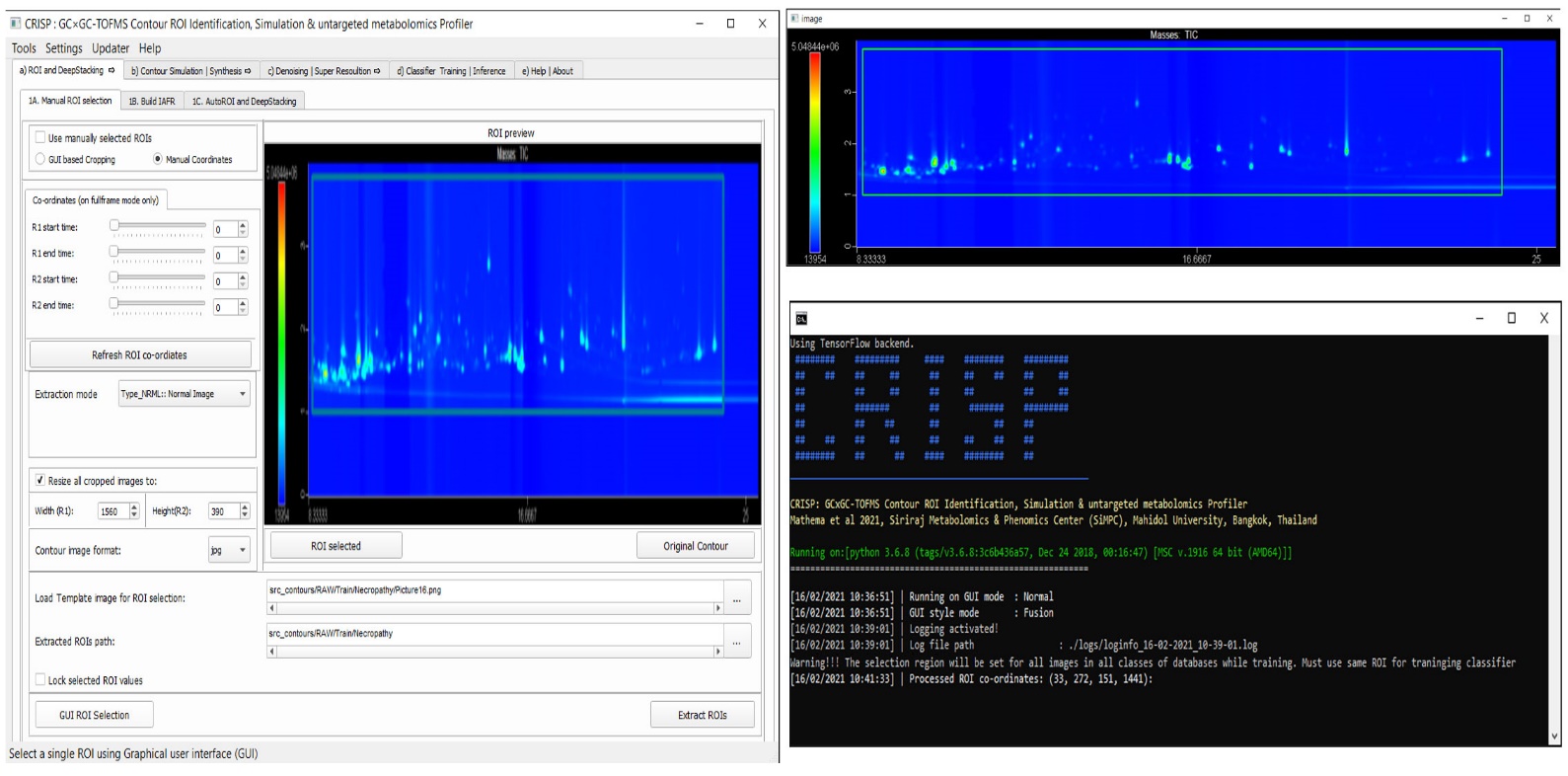
**Figure S2.** Single manual selection ROI selection. The screenshot of CRISP GUI and console screen showing example of a single manual ROI selection from whole GC×GC-TOFMS contour as indicated the boxed region in the RAW contour image. GC×GC-TOFMS, two-dimensional gas chromatography time-of-flight mass spectrometry; ROI, region of interest.


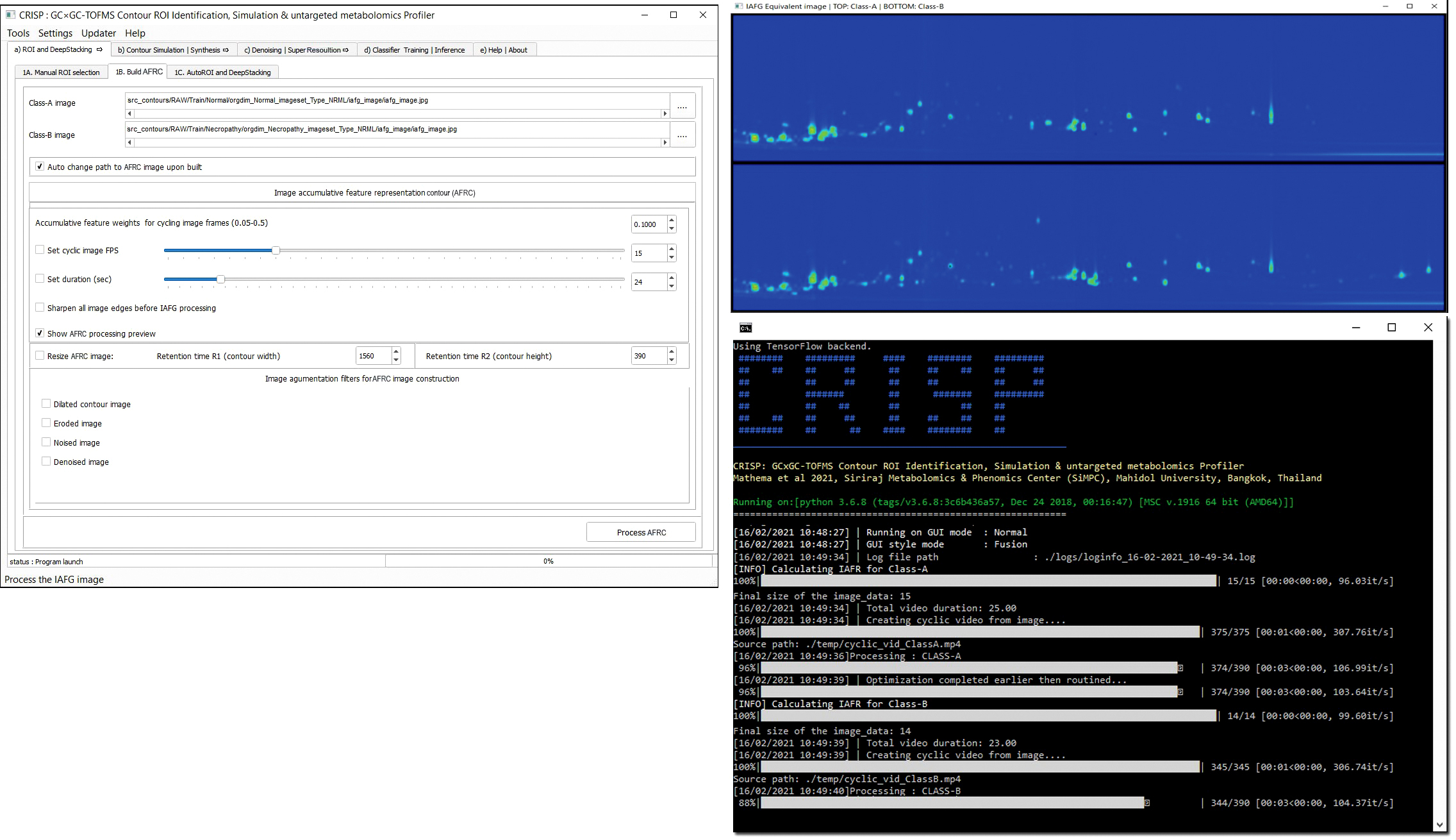


**Figure S3.** AFRC image construction from full frame GC×GC-TOFMS contour. Screenshot of CRISP module during construction of a single AFRC using default parameters for each of two groups (control vs ESRD/DM). AFRC, aggregate feature representative contour. GC×GC-TOFMS, two-dimensional gas chromatography time-of-flight mass spectrometry; ESRD/DM, end-stage renal disease with diabetes mellitus.


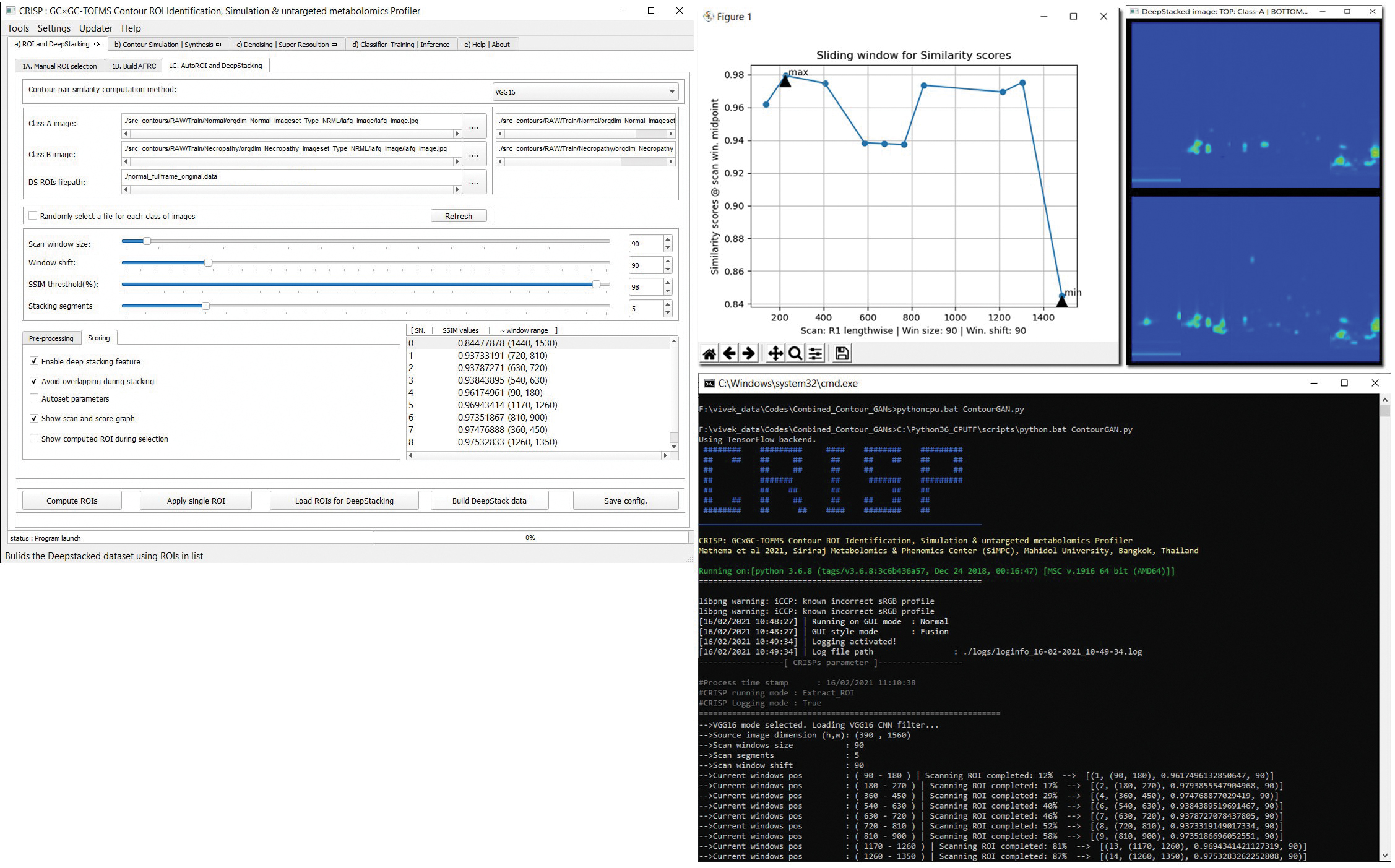


**Figure S4.** Deep stacking of ROIs. Screenshot of the typical use for CRISP deepstacking module showing semi-automatic identification and selection of multiple ROIs using the default VGG16 filter and given parameters for construction of feature enriched ROIs stacked (deepstacked) GC×GC-TOFMS contours dataset, which can be used for training the GAN simulation module or directly used for training classifiers if large numbers of samples are available. ROI, region of interest; GC×GC-TOFMS, two-dimensional gas chromatography time-of-flight mass spectrometry. ESRD/DM, end-stage renal disease with diabetes mellitus.


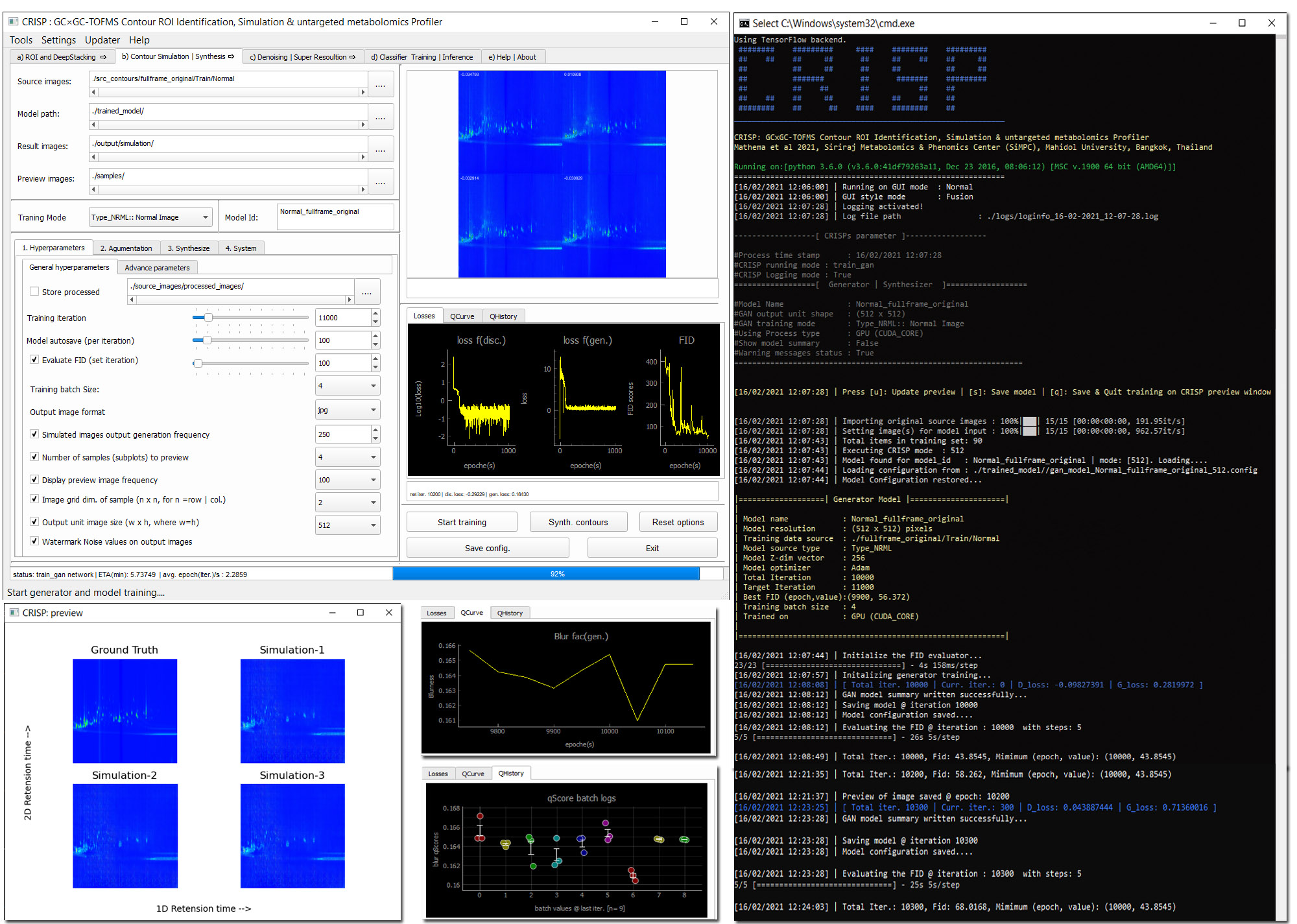


**Figure S5.** Contour simulation and generator training. The screenshot of CRISP module showing real-time generator training for GC×GC-TOFMS contour simulation. The graphs of generator, discriminator, and qScore are shown in real-time along with randomly selected real contour and user-defined number of simulated contours. The updates on console shows additional information of training progression including custom model summary. GC×GC-TOFMS, two-dimensional gas chromatography time-of-flight mass spectrometry.


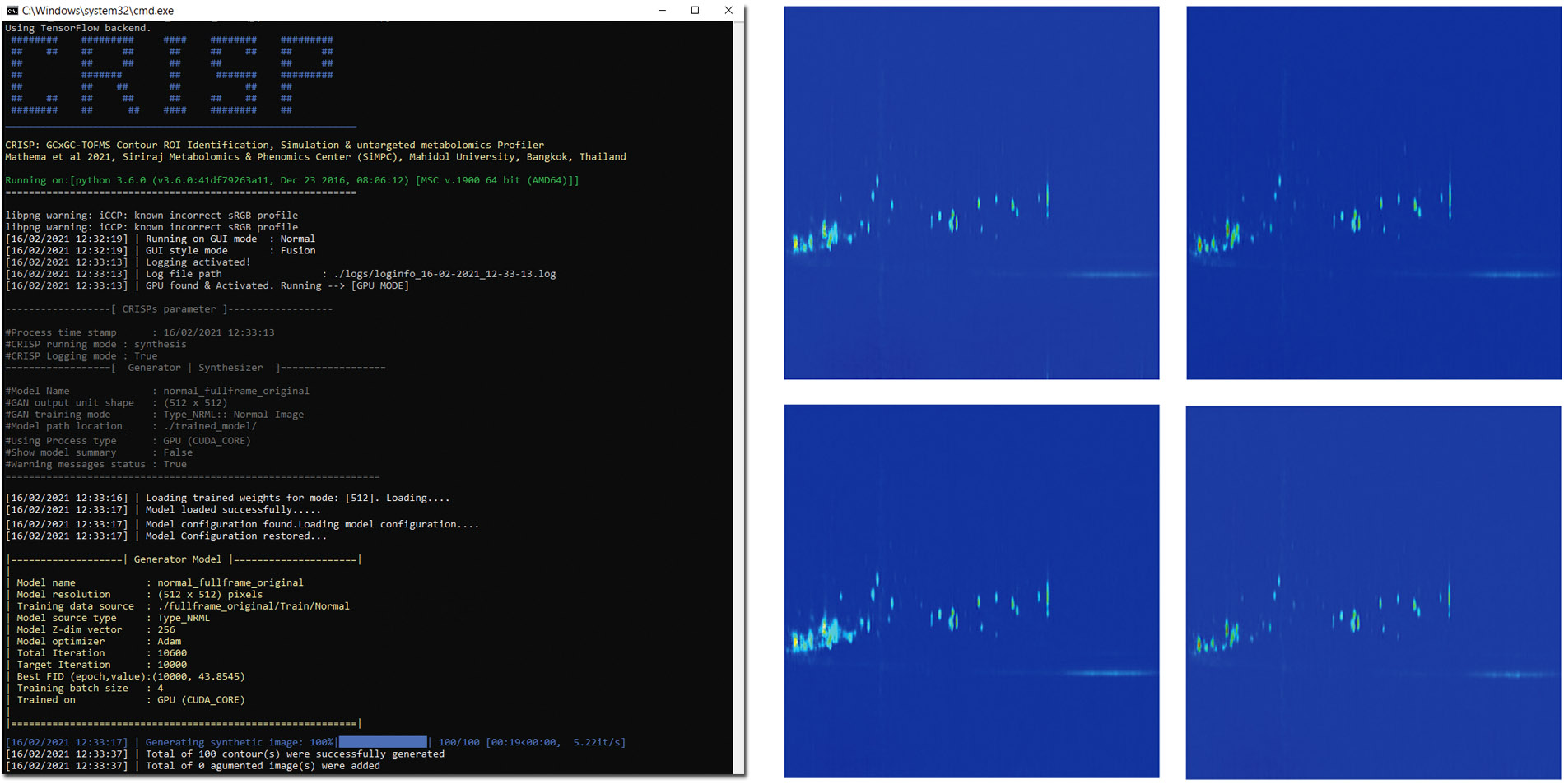


**Figure S6.** Synthesis of GC×GC-TOFMS contours using trained model. On left, screenshot of CRISP console showing synthesis of user-defined number of contours. On right, images of four synthetic contours obtained using trained generator which can be used for classifier training. Model training history and customized summary of trained model is displayed during each model launch. GC×GC-TOFMS, two-dimensional gas chromatography time-of-flight mass spectrometry


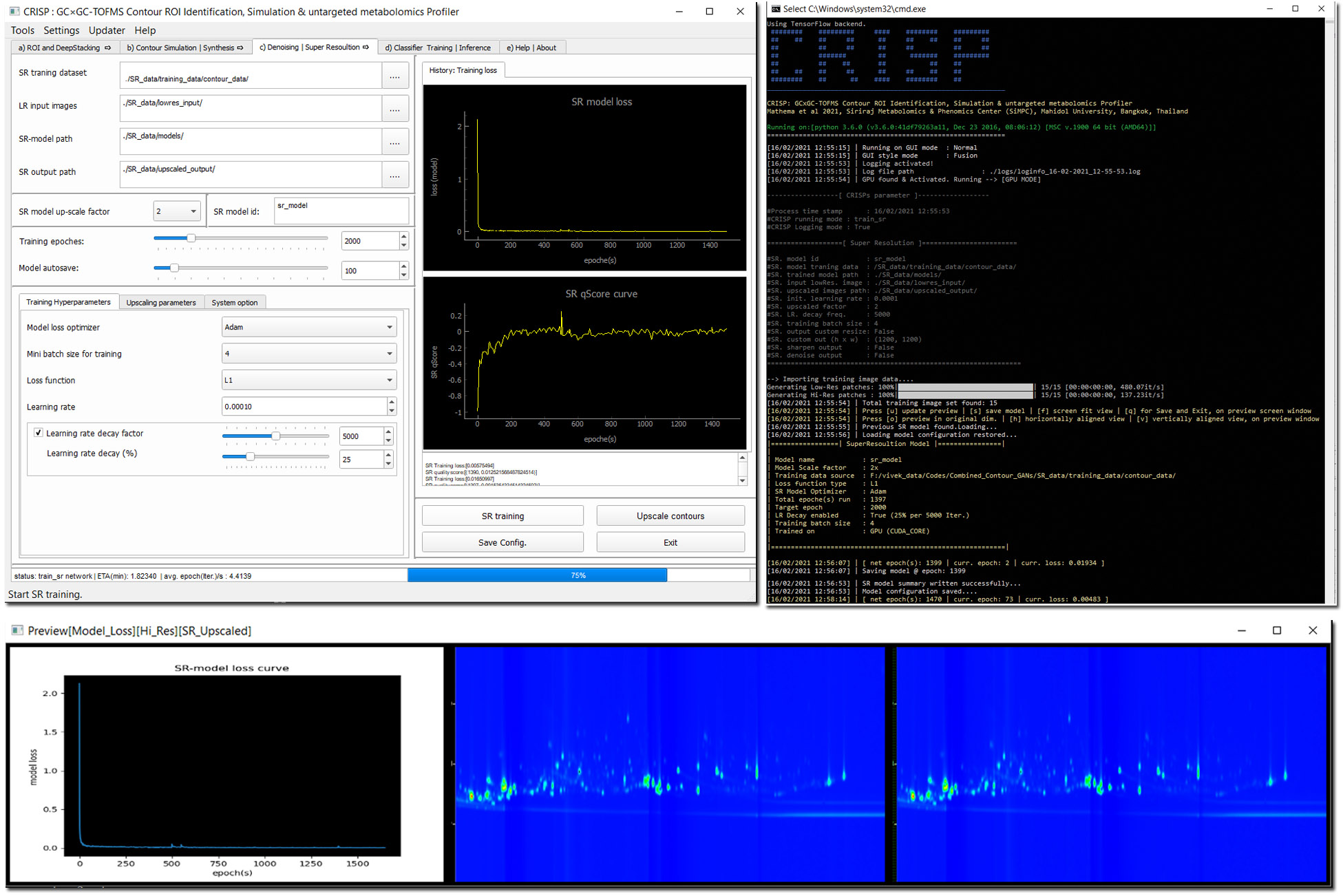


**Figure S7.** Superresolution model for contour quality improvement. The screenshot of the CRISP module ongoing training of GC×GC-TOFMS contour super resolution model for improvement of contour quality. The GUI shows real-time model loss along with qScore and preview of real as well as resolution-enhanced (2x) image during training session. The updates on console also shows detail information of training progression including custom model summary. GC×GC-TOFMS, two-dimensional gas chromatography time-of-flight mass spectrometry. GUI, graphical user interface.


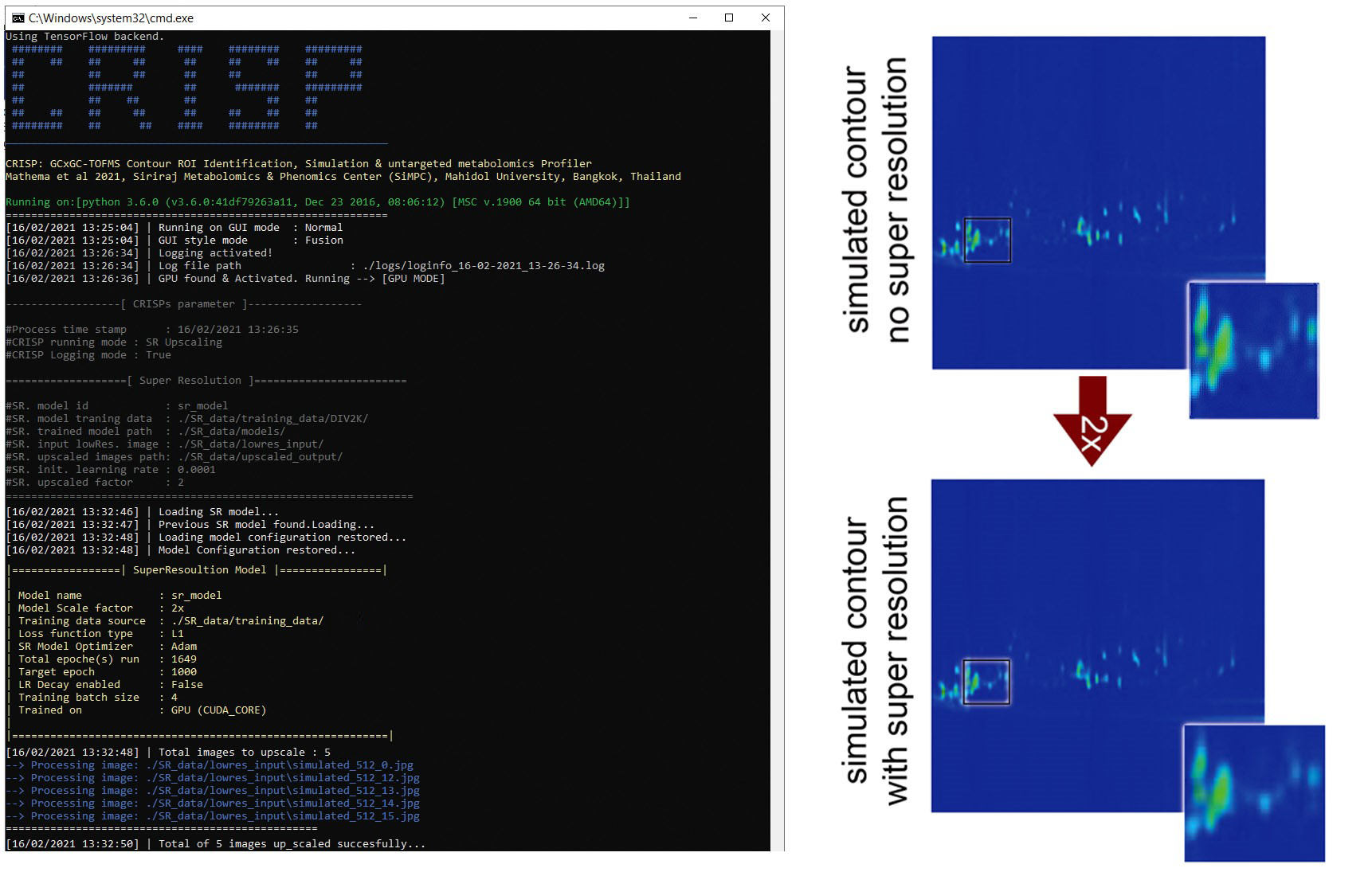


**Figure S8.** Super resolution enhancement of GC×GC-TOFMS simulated contour images. The screenshot showing ongoing image quality enhancement of simulated contour images based on the custom trained super resolution model. The left panel shows console information containing model summary and progression of the resolution enhancement process. The right panel shows a zoomed example of the improvement on simulated GC×GC-TOFMS contour resolutions based on custom trained CRISP super resolution model. The updates on console shows detailed information of trained model including model summary. GC×GC-TOFMS, two-dimensional gas chromatography time-of-flight mass spectrometry.


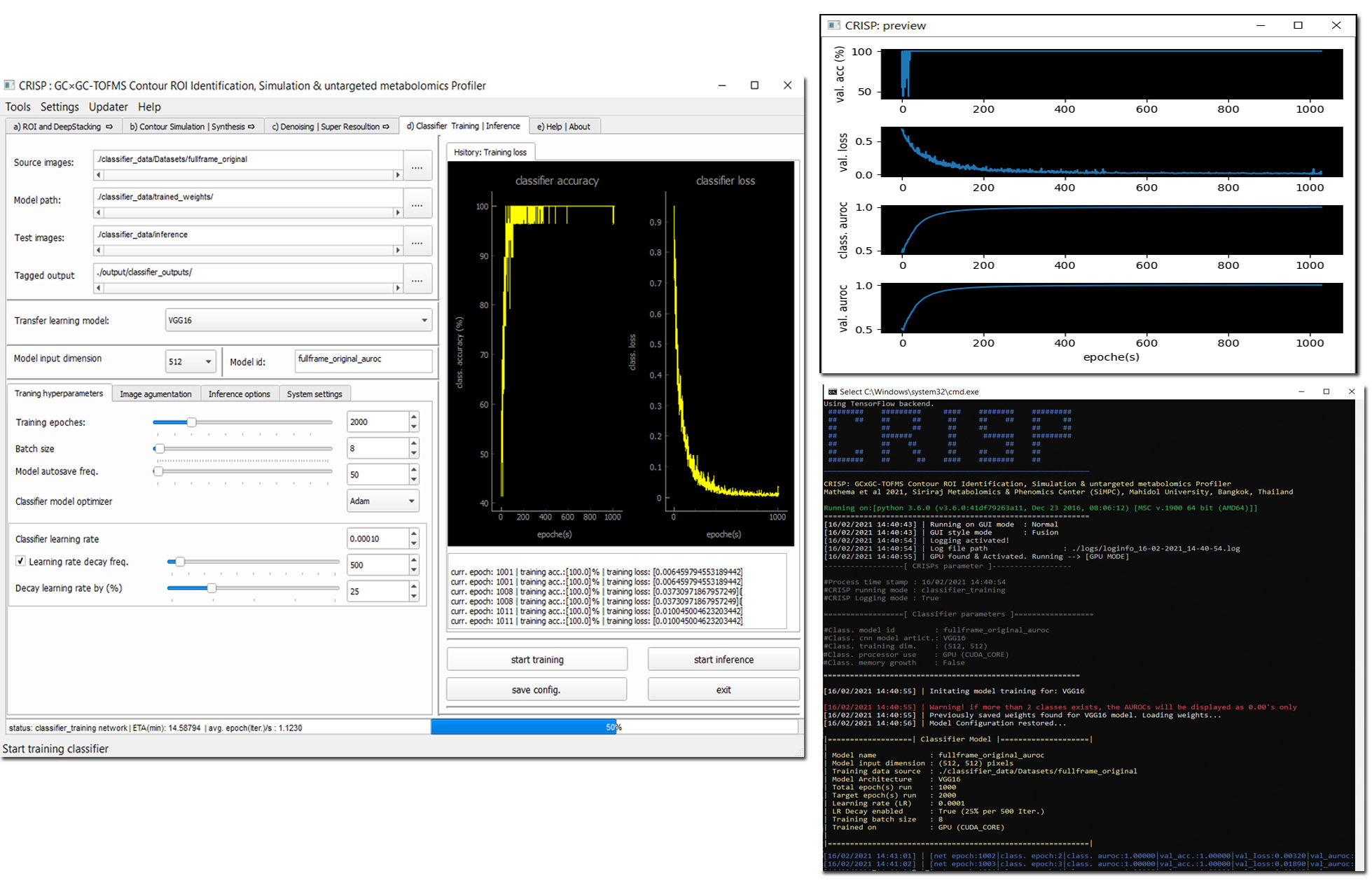


**Figure S9.** Classifier training using transfer learning. Screenshot of CRISP module for ongoing classifier training based on transfer learning method using VGG16 architecture. Real–time update graphs for model classification and validation accuracies, model losses and AUROC curves are displayed. The console screen displays the model summary and ongoing model training status. AUROC, area under the receiver operating characteristic.


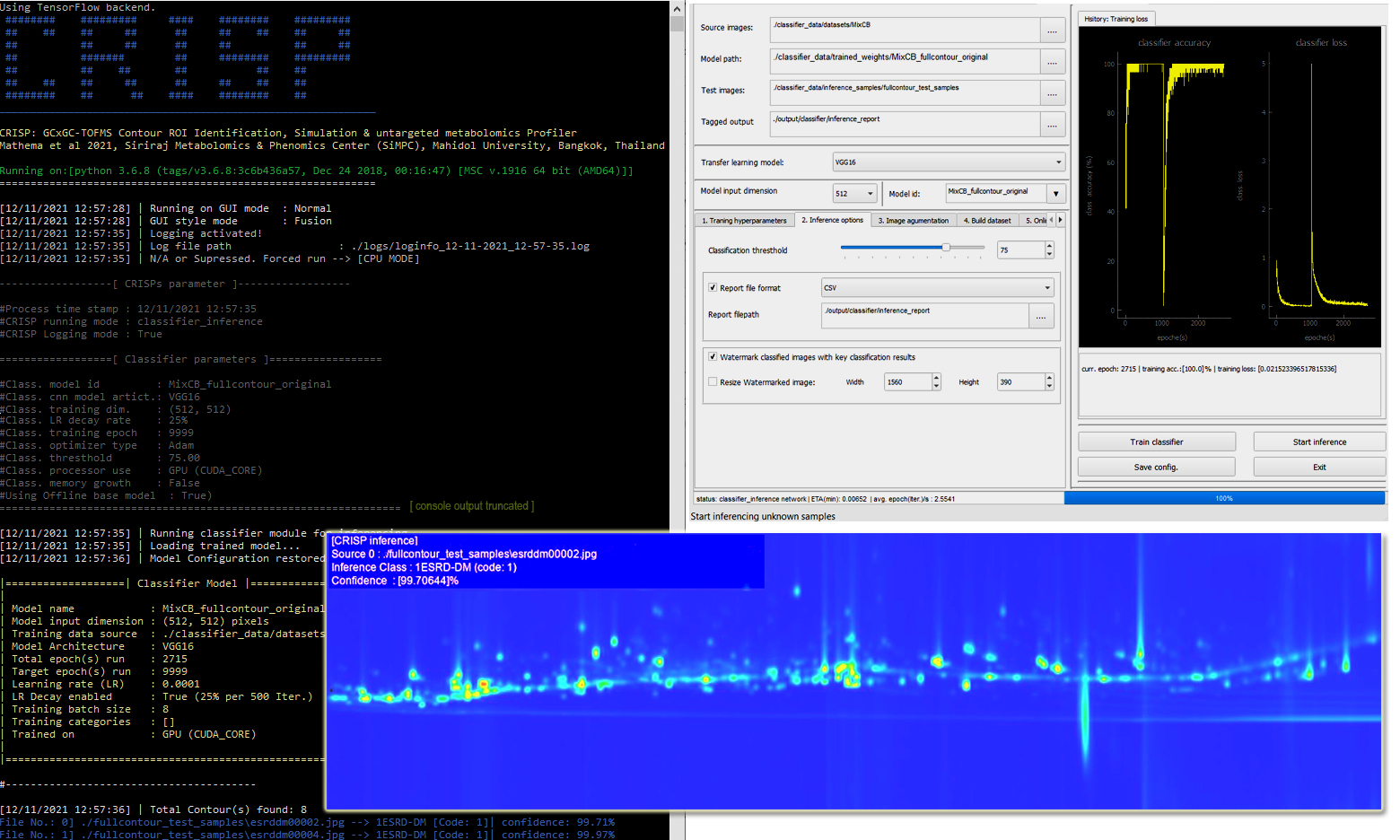


**Figure S10.** Unknown sample inference using trained classifier. Screenshot of the CRISP module showing inference results of unknown samples to predicted classes. The user can define the classification threshold and confidence of prediction to be watermarked on test samples. The result can be saved as text, CSV, or TSV files along with the watermarked contour images containing source name, predicted class and confidence of prediction. Model training history and customized summary of trained classifier model is displayed during each model launch. CSV, comma-separated values; TSV, tab-separated values.


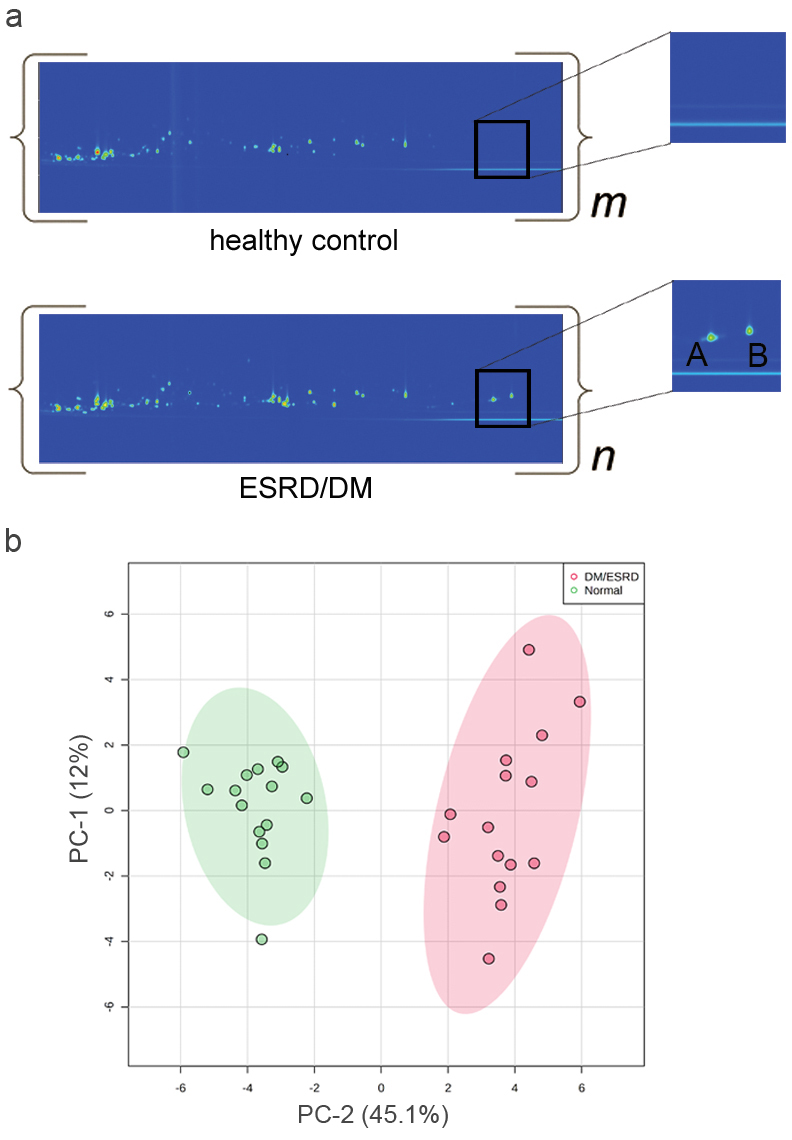


**Figure S11.** Contrasting feature between ESRD/DM and healthy control GC×GC-TOFMS contour feature. a) The contrasting features between GC×GC-TOFMS contour image of healthy control (top) and ESRD/DM (bottom). The metabolite A and B represents maltitol and lactitol, respectively b) The PCA results for randomly selected approximately 80% of ESRD/DM and healthy control samples showing difference in metabolite profile based on GC×GC-TOFMS raw data. ESRD/DM, end-stage renal disease with diabetes mellitus. GC×GC-TOFMS, two-dimensional gas chromatography time-of-flight mass spectrometry; PCA, Principal component analysis.
